# A Comparison of Models for Teaching Suturing and Surgical Skills to Dental Students

**DOI:** 10.1155/2024/3783021

**Published:** 2024-05-20

**Authors:** Michaelina Macluskey, Gavin Revie, Simon D. Shepherd

**Affiliations:** School of Dentistry, University of Dundee, Park Place, Dundee DD1 4HN, UK

## Abstract

Learning suturing skills is an important area of the undergraduate curriculum and ideally requires realistic and anatomically accurate surgical training models to prepare students for treating patients. Little is currently understood regarding which model might be perceived by students to be the best or which might most effectively facilitate their learning. The aim of this study was to compare four teaching models: a tabletop silicon dental model, a restricted access tabletop model, a traditional phantom head mounted model, and a Thiel cadaver. Student preferences were explored for each of the models. Following lecture and video-based teaching 67 fourth-year students attended a practical suturing teaching session followed by the second session more focused on the experience of cutting and suturing mucoperiosteal flaps. Forty-six students (67%) gave online anonymous feedback on the first session. The majority (95%) felt prepared to place a simple interrupted suture on a patient, and 88% felt confident to do so. Twenty-eight students (40%) provided feedback on the second session with 82% agreeing that they were prepared to cut a mucoperiosteal flap and 48% felt confident to do this for a patient. The cadaver model was rated as the best of the four models for both suturing and mucoperiosteal flap skills. These results support its use for teaching students to suturing and surgical skills. However, despite this teaching student-rated confidence to cut and suture flaps for a patient remains poor.

## 1. Introduction

Suturing is an essential skill in dentistry and providing a realistic training model to develop these skills can be challenging. Despite the relative importance of suturing skills, surgical training in undergraduate education does not always suitably prepare students for clinical practice following graduation [1]. As such, it is vitally important to understand which training resources might best facilitate student learning during their core training pathway.

The literature has many accounts outlining the teaching of suturing skills primarily to medical students with the use of dry and wet models [2]. There are also accounts of the use of reductant tissue [3], chicken skin [4], oranges [5], and silicon models [6]. For application to the practice of clinical dentistry, and to recreate the limited space of the oral cavity, models with restricted access have been developed [7]. Ideally an anatomically correct model would be used to teach these specific technical skills with the expectation of a smoother translation to the clinical chairside environment and a live patient.

The COVID-19 lockdown and staffing changes precipitated necessary innovation and evolution in teaching leading to the use of Thiel cadavers [8] as a suture model. Thiel models for suturing have been well received by our students. Students had been previously exposed to them for exodontia teaching [9]. Over many years in Dundee, several surgical training models have been adopted including porcine models, a commercially available flatbed model and more recently in-house manufactured silicon models allied to be spoke 3D printed model jaws [10]. These models can be mounted in phantom heads and allow students to cut and raise flaps and subsequently suture them back in place providing a more realistic understanding of the anatomical restrictions of the oral cavity, especially in dentate cases. To enhance the learning experience, the use of a video resource to prepare the students in advance of the teaching is advocated [2, 9, 11] and has been recommended as part of good educational framework [12]. Video-assisted self-monitoring has been shown to be effective for teaching suturing skills to dental students using the plan-do-check-act (PDCA) cycle [13]. It has also been shown that students use open access video resources to supplement teaching within their curriculum [14].

The primary aim of this study was to determine which model our students perceived as best preparing them to cut and raise a mucoperiosteal flap and to place a suture in a live patient. A secondary aim was to determine if the teaching had adequately prepared them for chairside clinical practise by an objective assessment of their suturing skills. Our objectives were to deliver hands-on practical surgical skills teaching using different surgical training models, to understand/evaluate student self-report views/perceptions on which of the surgical training models they felt best supported their learning, to provide teaching and experience of mucoperiosteal flaps, and to determine whether this teaching prepared them for an objective assessment of suture skills.

## 2. Materials and Methods

Ethical approval was granted for this prospective study (UOD-SREC-SDEN-2022-023). All fourth-year dental students (*n* = 67) were notified by email in advance of the planned teaching with a participant information leaflet outlining what the study involved. Prior to the practical teaching session, students received lecture-based teaching on surgical principles including suturing, suture materials, instrumentation, and flap design for different surgical applications including third molars, retained root removal, and periradicular surgery. A video resource demonstrating the placement of simple interrupted sutures on a flatbed model was made available on the virtual learning environment (VLE) and the students were directed to view this in advance of the first teaching session.

**Figure 1:**
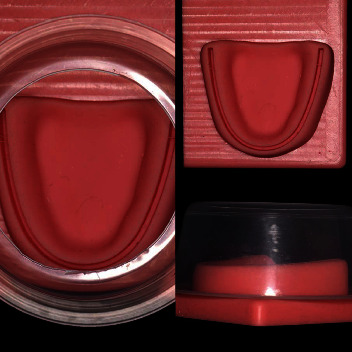
Flatbed and restricted models: left—top view of restricted version, bottom right—side view restricted model, and top right—unrestricted flatbed.

**Figure 2:**
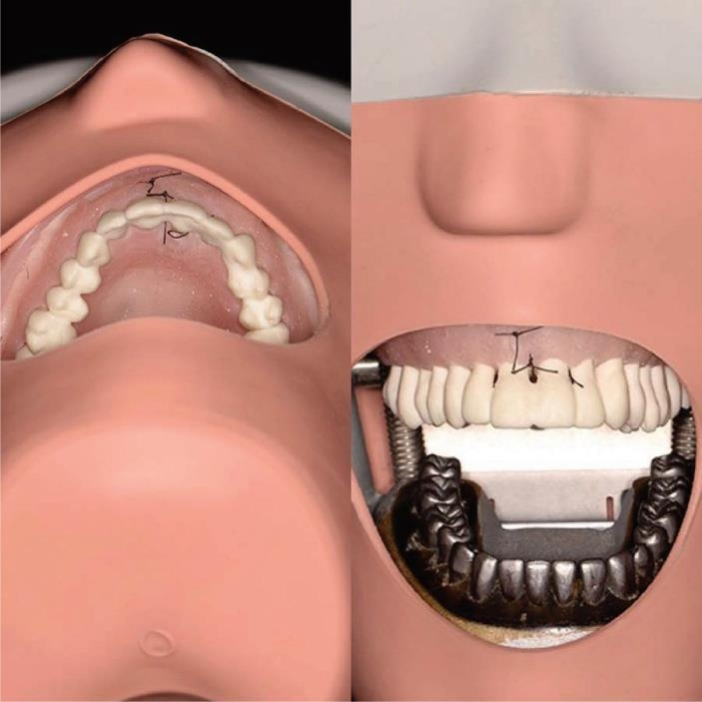
Dundee suture model in situ in the phantom head model (note: metal lower arch to replicate lower arch anatomy, not used for suturing).

Written consent to participate was provided at the start of each of the sessions. The same two experienced members of staff delivered all teaching on a 1 : 4 staff–student ratio. The first 90-min session focused on suture technique using four models; a commercially available tabletop silicon dental model (Gaetooely), the same model with a plastic cone positioned over the dental arch to restrict access (Figure 1), our traditional phantom head mounted model (Figure 2), and a Thiel cadaver.

Staff demonstrated the technique of a simple interrupted suture sequentially on each of the four models and then observed each of the students providing feedback in real-time. The second session took place a few weeks later and focused on mucoperiosteal flap design with staff demonstrating the range of flaps that could be used for different scenarios and how to reposition and suture the flaps using two models, the phantom head mounted and Thiel models. Teaching on the Thiel cadavers was on a 1 : 1 staff : student ratio which afforded each student an explanation of anatomical complications. The students started by suturing extraoral skin to mimic the flat tabletop model, moved to easily accessible intraoral sites and then were able to place sutures in difficult to reach areas that they may not have been afforded an opportunity to do on a patient, such as the floor of mouth or the soft palate. They could experience the difference in thickness and textures at different anatomical sites that they had not appreciated. The sessions also offered further opportunity for supervised and instructed suture practice on the three other models.

All students were invited by email to complete an anonymous online survey after each session. Questions included perceived student confidence of suturing using each of the models using a Likert scale [15], free text comments, and ranking each model in order of their preference for the one they felt most prepared them to treat patients. The ranking was tested using a Friedman's ANOVA test followed by a Bonferroni corrected pairwise comparison of models. The survey questionnaire is shown in Table 1.

After the teaching sessions, all models were made freely available in the department for the weeks prior to the suturing assessment for students to practise. The formal assessment employed the same phantom head mounted model used in previous years (Figure 2) using an objective validated checklist that was made available to students in advance of the assessment [16, 17]. As this was a formative assessment, students could attempt the assessment repeatedly until they had attained a satisfactory performance.

## 3. Results

All fourth-year students (*n* = 67) took part in the teaching and 46 students (67%) provided feedback on the first session, 38% were males and 61% were females with 24% having a previous degree and in 15% the degree was in an allied health care profession. The responses to the questionnaire as outlined in Table 1 are shown in Table 2. Ninety-eight percent found the video resource useful in advance of the teaching session with free text comments stating that they felt the video helped to prepare them for the practical teaching. However, they did not feel sufficiently prepared to place a suture in a restricted model or on a patient after having just watched the video without the benefit of the added direct practical teaching. Student reports suggest that the video was better than written information, diagrams, or illustrations to prime them for the class.

Student comments:

*“The video made me feel more prepared for the practical session. However, I would not have felt prepared to place a suture on a patient after watching the video alone.”*

*“It was useful as far as identifying the instruments and explaining why they are used the way they are and what the correct grips are for those instruments. Where it lacked was in its representivity (sic) with regards to a restricted environment. Sutures being placed in a phantom head would have prepared us for the need to move the needle holder forceps closer to the knot when tightening the suture.”*

*“It was a useful aid to familiarise yourself with the general technique before trying it yourself in the lab and then also following the session to consolidate the technique tips given throughout the session as you could replay it and slow it down to watch each part of the suture process.”*

*“Useful as a contextualisation video and if I really needed to, I could learn from it, but nothing can beat seeing it done in real life and being able to ask questions.”*

After the first session, 95% felt that the teaching had prepared them to place a suture on a patient. Confidence in placing a suture was high with 96% being confident to place a suture on the commercial silicon model, 96% on the phantom head mounted model and 91% on the cadaver model. When asked if they felt confident to place a suture on a live patient 88% agreed.

Forty-six of the original 67 participants provided ratings for all the different model types and were included in Friedman's ANOVA. Inspection of the data revealed some potential issues that needed to be addressed prior to conducting the analysis. Ten of the participants apparently misinterpreted the task instructions and did not utilise the correct ranking system to sort the models from perceived best (1) to worst (4). Rather, it appears respondents used the 1–4 ranking as a model rating (rather than ranking) system, reusing some scores when they felt items were tied. As an example, one participant gave every model a score of 1. As long as the data are ordinal (i.e., 1 = best rating), Friedman's ANOVA can still analyse these results. However, the possibility that participants had just completely misinterpreted the instructions could not be discounted, so a sensitivity analysis was conducted to determine the effect of removing participants who had not followed the instructions exactly. In all cases, there was no change to the overall pattern of results by excluding the 10 probematic cases, with the overall Friedman's ANOVA *p*-value shifting to be very slightly more significant (by 0.000057) if all 10 erroneous cases were dropped demonstrating that the erroneous use of the ranking system had negligible impact on the results. As such, the results are reported here with all participants included.

**Figure 3:**
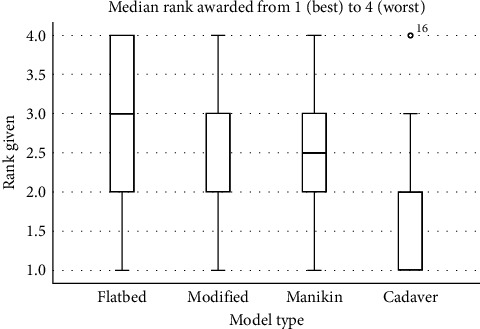
Rating of the four models from 4 (worst) to 1 (best) by the students (*n* = 46) using a Friedman's ANOVA (*p*=0.00006).

Students rated the cadaver as the best model and the flatbed the least favourable (Figure 3). A Friedman's ANOVA found a significant difference in the average score awarded to the models (*χ*^2^_F_ (3) = 22.159, *p*=0.00006) and a post hoc pairwise comparison of models found that the cadaver was rated better than the flatbed, modified flatbed and manikin models (Bonferroni adjusted *p*-values of 0.00006, 0.00028, and 0.00044, respectively). None of the remaining comparisons (phantom head vs. modified flatbed, phantom head vs. flatbed, modified flatbed vs. flatbed) between the models were close to statistical significance (all adjusted *p*-values circa 1).

Free text comments on the tabletop silicon model suggested that the students found this to be relatively easy as there were no restrictions on space, so it was good to master the basic technique but not very realistic. The restricted access model added another layer of complexity making it more challenging offering an indication of the kind of space restriction suturing in the mouth will bring. Our traditional phantom head mounted silicon model added more realistic challenges of a confined space, some semblance to dento-alveolar anatomy and the requirement to overcome the obstructions when suturing around the teeth. However, the thickness of the silicon reportedly made it difficult to handle, push the needle through, and the elasticity meant that the wound tended to spring open. Students certainly found this more challenging.

Student comments:

*“The silicone is thick and less realistic”*.

*“Silicone kept springing apart”*.

*“Having the phantom head model with actual contact points allowed me to gain good practice before progressing onto the cadaver. As the cadaver was fully dentate this made access and finding landmarks much more realistic.”*

*“The phantom head model I could clearly see where I needed to adjust my technique.”*

Students may be unsurprisingly found the cadaveric model the best with the most accurate anatomy. Reports mostly suggested that the tissues were more realistic than the silicon models; however, this was not universally welcomed as some students did not find the tissues sufficiently robust to accommodate their technique flaws. A few commented on it being unpleasant to touch.

Student comments:

*“Silicone is better as it is easy*, *but the cadaver gives it a real-lifescenario”*.

*“The elasticity of the tissues in the cadaver more closely simulated that to which we would encounter on real patients. It was also better for practising access as you had to deal with structures such as the tongue or increased cheek thickness which wasn't present on the silicone model.”*

After the second teaching session, 82% felt that they had sufficient knowledge and skills to cut a flap, 86% felt confident to cut and raise a flap on the phantom head mounted model, and 92% would be confident to suture the flap afterward on this model. Most (86%) felt confident to cut and raise a flap in the cadaveric model and 93% were confident to suture the flap afterward. However, only 48% felt confident to cut and raise a flap on a patient while 85% felt confident to suture a flap on a patient.

Seventy-eight percent felt that the cadaver model was better than the phantom head model for cutting flaps as it was anatomically accurate with realistic tissue handling although the phantom head models were useful to practise the technique. Some students said that the silicon was too thick and elastic, and a few others said that the cadaver mucosa was too flimsy and liable to tear.

Student comments:

*“Tissue handling was as close to a live patient as possible. Tissues were more prone to tearing which forces more careful use of suture needles.”*

*“Cadaver is flimsy.”*

Students appreciated that the cadaver model allowed them to visualise important anatomical structures like the mental foramen when working in the lower premolar region and appreciate the difficulty of suturing around and between teeth especially when suturing onto the palate. Students liked that staff were able to demonstrate advanced techniques such as closure of oroantral communication and demonstrate periosteal release for buccal advancement flaps.

Student comments:

*“The elasticity of the tissues in the cadaver more closely simulated that to which we would encounter on real patients. It was also better for practising access as you had to deal with structures such as the tongue or increased cheek thickness which wasn't present on the silicone model.”*

*“I also thought that visualising the mental nerve bundle and the lingual nerve was very beneficial.”*

A few weeks after the teaching the suturing assessment was completed by 67 students with a mean percentage and standard deviation of 89.9% ± 9.87. Nine students were not successful at the first attempt so were offered revision. All were successful at the second attempt.

## 4. Discussion

Suturing is an essential skill for dental students to acquire before graduation and must be assessed to be at the level of a safe beginner [18]. Many suture models are available to teach this skill and our institution has used various models over the years, but many are not realistic enough to adequately prepare our students for real patients. It has been reported that the acquisition and quality of basic suturing sills are influence by the model used [19]. COVID-19 imposed necessary changes and our models evolved to the Thiel cadavers. Subsequent student feedback was very positive and their performance on an objective assessment was good suggesting that this change may have been an improvement on previous teaching [9]. To test this more thoroughly, we asked our students to try four models to see which of these they felt was better to prepare them for suturing on patients.

The results confirm our previous findings that the cadaver model is better appraised by the students as a model for suturing and supports its use for teaching mucoperiosteal flaps [9]. The students were positive about the use of the video in preparation for the teaching which is in keeping with other reports [9, 12–14]. By increasing the degree of difficulty and anatomical realism of the models, we hoped to help the students learn the basic technique and then understand, and be able to apply, modifications to accommodate the space limitations encountered in the oral cavity. Students found the tabletop model relatively easy as a starting point which worked well to master the basic techniques with the introduction of the restricted model giving them further insight into the difficulty of operating in a confined space. The phantom head mounted model was more challenging still, requiring students to negotiate teeth, contact points, and buccal tissues. However, the consistency of the silicone proved to be challenging and the elasticity meant that wound approximation was not as easy as the students might have liked. Staff observed that the elasticity of the silicone brought other attendant learning challenges. That is that students were able to, despite instruction to the contrary, lift the palatal silicone “mucosa” to facilitate suturing at this site. This factor was explained and reinforced during the use of the cadaver model as raising the palatal mucosa is clearly not feasible in the live patient. Unfortunately, this remained a residual common fault identified during the assessment procedure, and which poses questions about (despite the reported advantages) the transferability of the silicone model version to live clinical practice.

If anything, the cadaveric model demonstrated how real mucosa handled both with suturing and when cutting and raising mucoperiosteal flaps allowing the students to appreciate that the phantom head model was the most difficult model to use. A potential lack of insight into how poor student technique led to mucosal tearing in the cadaver may have led to the criticisms of “flimsy” tissues, not least because teachers found the mucoperiosteum surprisingly robust considering the embalming process.

It was important to allow the students access to the models to continue to practise prior to the assessment asretention of such skills is poor otherwise [20]. Unfortunately, this same opportunity could not be extended to the cadavers secondary to access limitations and availability of staff to supervise at this site. This cohort of students performed on a par with previous cohorts using the same model and checklist for the assessment [9]. The main difference was that in previous years, this assessment formed part of a larger multistation summative assessment but on this occasion, it was a standalone formative assessment. Not being a high-stakes assessment may explain or be contributory to why so many students failed at the first attempt.

Confidence in placing a suture was relatively high, at least when measured in close temporal relationship with the delivery of the teaching. What is unknown is whether that confidence would transfer to confidence when needing to place a suture in a real patient and with a time interval following the teaching. Additionally, the correlation with reported self-confidence and actual objectively observed ability in undergraduate dental students for this skill is unknown, whether that be on model or a live patient.

A smaller part of this work was to determine if these models enhanced our minor oral surgery teaching, and more specifically, on mucoperiosteal flap design. Students again rated the cadaveric model higher than the phantom head mounted model as they felt it was more realistic and had easier handling properties. Prior lecture-based teaching along with the second practical session offered an opportunity to consolidate knowledge and skills in a hands-on practical class on cutting and raising flaps. Previous studies have suggested that students lack confidence in this area [21, 22]. Our results tend to support this. Most telling was the low confidence reported in response to being asked if they felt confident to raise a flap at any given site. However, this feedback was from only one 90-min teaching session, and it may be postulated that increased exposure to the cadaver model may help improve student confidence; however, this was not feasible within the restrictions of the timetable and access to the anatomy facilities. Additionally, the very poor response rate to the second session survey makes any extrapolation of these findings uncertain.

Undergraduate dental student experience of surgery across the United Kingdom is quite limited [23, 24] and we have shown that at the end of final year students still lack confidence in surgery [25]. The potential consequences and wider health service cost could be great if poor clinician confidence influences referral patterns with resultant impact on specialist services, patient waiting times and secondary care treatment costs [26]. One major difficulty is how to provide enough hands-on operating for students to gain sufficient experience for proficiency, without impacting on their experience of other disciplines, and for this to have a durable effect so they might continue to operate after graduation. The next stage for this work would be to determine whether students have acquired transferable and long-lasting chair side suturing skills.

The limitations of this study were the small sample size and the relatively low response rate, especially to the second teaching session, so the findings must be interpreted with this caveat. Conducting large multicentre studies would not be possible as there a too few centres that use Theil embalmed cadavers for teaching dental students, we believe we are the only school doing so in the United Kingdom. The same two staff provided all the teaching and also acted as examiners for the assessments so potential criticism could include the introduction of an element of bias. However, it could be argued that this made for a more consistent teaching and assessment experience. One major disadvantage of the teaching approach is the very labour-intensive staff costs to retain the favourable small group staff : student ratio. It should also be emphasised that the positive numerical ratings given to the cadaveric models reflect what students *say* rather than any kind of objective measure. These values could be distorted by various factors unrelated to the teaching quality for example the perceived prestige of a novel teaching method or responding in a way that they think we want to hear. Future research might seek to understand whether student learning can be as well delivered with additional video resources, simple models for home practice, video recording and independent review, additionally review by peers or staff, followed up with shorter intense supervised sessions on the cadavers to consolidate skills. Comparisons between cadaveric and other teaching methods could be conducted using less overt questions to obscure the research objective from participants. It may even be possible to use a double-blind design where those collecting ratings do not know what model the student was exposed to, and the student does not know what models other students used.

## 5. Conclusions

There are numerous models available for teaching suturing skills to students, each with advantages and disadvantages. Student preparedness for clinical practice is paramount and so understanding which teaching resources might best facilitate student learning within the constraints of the curriculum is important. The Thiel cadaver model has been well received by our dental students for teaching minor oral surgery with suturing and mucoperiosteal flap skills. The students have generally rated them as superior to silicon benchtop models and phantom head mounted models addressing this study's primary aim. This is understandable given that they afford a greater degree of realism for tissue handling and anatomical accuracy giving a greater understanding of the challenges of suturing in patients mouths. More work needs to be done to prepare students to cut and raise mucoperiosteal flaps in a live patient as this has been identified as an area of need. Future research into this area might explore associations with self-report confidence in suturing with objective assessments in the clinical setting.

## Figures and Tables

**Table 1:** The questions and coding for the student questionnaire for sessions 1 and 2, omitting free-text responses.

Questions	Score and coding
*Session 1*	
(1) I found the online suture video useful for my learning (2) The teaching that I have received has given me sufficient knowledge and skills to place simple interrupted sutures (3) I feel confident that I could place interrupted sutures on a flatbed model (4) I feel confident that I could place interrupted sutures in a phantom head mounted model (5) I feel confident that I could place interrupted sutures in a cadaver model (6) I feel confident that I could place interrupted sutures in a patient	Likert score 1–5

(7) Can you rank the models in the order from the best (1) to the worst (4)	1—best, 4—worst

*Session 2*	
(8) The teaching that I have received has given me sufficient knowledge and skills to cut and raise a flap (9) I feel confident to cut and raise a flap on a phantom head mounted model (10) I feel confident that I could place interrupted sutures in a phantom head mounted model (11) I feel confident to cut and raise a flap on the cadaver model (12) I feel confident that I could place interrupted sutures on the cadaver model (13) I feel confident that I could now cut and raise a flap on a patient (14) I feel confident that I could place interrupted sutures in a patient	Likert score 1–5

(15) Can you rank the models in the order from the best (1) to the worst (2)	1—best, 2—worst

(16) I feel confident to cut and raise a mucoperiosteal flap on a patient for the removal of an impacted lower third molar (17) I feel confident to cut and raise a mucoperiosteal flap on a patient for the removal of the root 14 (18) I feel confident to cut and raise a mucoperiosteal flap on a patient for the removal of a root for tooth 46 (19) I feel confident to cut and raise a mucoperiosteal flap on a patient at any site	Likert score 1–5

Likert score: 1—strongly disagree; 2—disagree; 3—neither disagree nor agree; 4—agree; and 5—strongly agree.

**Table 2:** Responses to questions in Table 1 are given as a percentage of the total number of students responding (*n* = 46) for session 1 questions 1–6 and for session 2 (*n* = 28) questions 8–14 and 16–19.

Question	SA	A	N	D	SA
(1) I found the online suture video useful for my learning	58.7	39.1	2.2	0	0
(2) The teaching that I have received has given me sufficient knowledge and skills to place simple interrupted sutures	76.1	19.6	0	4.3	0
(3) I feel confident that I could place interrupted sutures on a (silicone) flatbed model	82.6	13	2.2	2.2	0
(4) I feel confident that I could place interrupted sutures in a silicon phantom head mounted model	56.5	39.1	0	4.3	0
(5) I feel confident that I could place interrupted sutures in a cadaver model	60.9	30.4	4.3	4.3	0
(6) I feel confident that I could place interrupted sutures in a patient	26.1	58.7	2.2	8.7	4.3
(8) The teaching that I have received has given me sufficient knowledge and skills to cut and raise a flap	64.3	32.1	0	3.6	0
(9) I feel confident to cut and raise a flap on a silicon phantom head mounted model	42.9	39.3	7.1	10.7	0
(10) I feel confident that I could place interrupted sutures in a silicon phantom head mounted model	46.4	39.3	0	14.3	0
(11) I feel confident to cut and raise a flap on the cadaver model	60.7	32.1	0	7.1	0
(12) I feel confident that I could place interrupted sutures on the cadaver model	32.1	53.6	0	14.3	0
(13) I feel confident that I could now cut and raise a flap on a patient	53.6	39.3	0	7.1	0
(14) I feel confident that I could place interrupted sutures in a patient	7.1	39.3	32.1	14.3	7.1
(16) I feel confident to cut and raise a mucoperiosteal flap on a patient for the removal of an impacted lower third molar	0	42.9	46.4	3.6	7.1
(17) I feel confident to cut and raise a mucoperiosteal flap on a patient for the removal of the root 14	3.6	60.7	25	3.6	7.1
(18) I feel confident to cut and raise a mucoperiosteal flap on a patient for the removal of a root for tooth 46	0	50	39.3	7.1	3.6
(19) I feel confident to cut and raise a mucoperiosteal flap on a patient at any site	0	28.6	39.3	25	7.1

SA, strongly agree; A, agree; N, neither agree nor disagree; D, disagree; and SD, strongly disagree.

## Data Availability

The quantitative data used to support the findings of this study are available from the corresponding author upon request.
